# Adagrasib, a KRAS G12C inhibitor, reverses the multidrug resistance mediated by ABCB1 in vitro and in vivo

**DOI:** 10.1186/s12964-022-00955-8

**Published:** 2022-09-14

**Authors:** Yuchen Zhang, Cheukfai Li, Chenglai Xia, Keneth Kin Wah To, Zhixing Guo, Chongyang Ren, Lingzhu Wen, Fang Wang, Liwu Fu, Ning Liao

**Affiliations:** 1 School of Medicine, South China University of Technology, Guangzhou, 510006 Guangdong Province China; 2 Collaborative Innovation Center for Cancer Medicine; State Key Laboratory of Oncology in South China, Guangdong Esophageal Cancer Institute; Sun Yat-Sen University Cancer Center, Guangzhou, 510060 China; 3 Department of Breast Cancer, Department of Surgery, Guangdong Provincial People’s Hospital, Guangdong Academy of Medical Sciences, Guangzhou, 510080 Guangdong Province China; 4 Foshan Women and Children’s Hospital Affiliated to Southern Medical University, Foshan, 528000 China; 5 School of Pharmacy, The Chinese University of Hong Kong, Hong Kong, 999077 China

**Keywords:** Adagrasib (MRTX849), Multidrug resistance, ABC transporters, KRAS mutation, Cancer chemotherapy

## Abstract

**Background:**

Multidrug resistance (MDR) is a complex phenomenon that frequently leads to chemotherapy failure during cancer treatment. The overexpression of ATP-binding cassette (ABC) transporters represents the major mechanism contributing to MDR. To date, no effective MDR modulator has been applied in clinic. Adagrasib (MRTX849), a specific inhibitor targeting KRAS G12C mutant, is currently under investigation in clinical trials for the treatment of non-small cell lung cancer (NSCLC). This study focused on investigating the circumvention of MDR by MRTX849.

**Methods:**

The cytotoxicity and MDR reversal effect of MRTX849 were assessed by MTT assay. Drug accumulation and drug efflux were evaluated by flow cytometry. The MDR reversal by MRTX849 in vivo was investigated in two ABCB1-overexpressing tumor xenograft models in nude mice. The interaction between MRTX849 and ABCB1 substrate binding sites was studied by the [^125^I]-IAAP-photoaffinity labeling assay. The vanadate-sensitive ATPase assay was performed to identify whether MRTX849 would change ABCB1 ATPase activity. The effect of MRTX849 on expression of ABCB1 and PI3K/AKT signaling molecules was examined by flow cytometry, Western blot and Quantitative Real-time PCR analyses.

**Results:**

MRTX849 was shown to enhance the anticancer efficacy of ABCB1 substrate drugs in the transporter-overexpressing cells both in vitro and in vivo. The MDR reversal effect was specific against ABCB1 because no similar effect was observed in the parental sensitive cells or in ABCG2-mediated MDR cells. Mechanistically, MRTX849 increased the cellular accumulation of ABCB1 substrates including doxorubicin (Dox) and rhodamine 123 (Rho123) in ABCB1-overexpressing MDR cells by suppressing ABCB1 efflux activity. Additionally, MRTX849 stimulated ABCB1 ATPase activity and competed with [^125I^]-IAAP for photolabeling of ABCB1 in a concentration-dependent manner. However, MRTX849 did not alter ABCB1 expression or phosphorylation of AKT/ERK at the effective MDR reversal drug concentrations.

**Conclusions:**

In summary, MRTX849 was found to overcome ABCB1-mediated MDR both in vitro and in vivo by specifically attenuating ABCB1 efflux activity in drug-resistant cancer cells. Further studies are warranted to translate the combination of MRTX849 and conventional chemotherapy to clinical application for circumvention of MDR.

**Video Abstract**

**Supplementary Information:**

The online version contains supplementary material available at 10.1186/s12964-022-00955-8.

## Background

Chemotherapy is an effective treatment for metastatic tumors. However, multidrug resistance (MDR) is one of the major obstacles to successful chemotherapy. It refers to the development of resistance of tumor cells to anticancer drugs with different structures and mechanisms[1]. MDR is caused by multifactorial mechanisms, including enhancement of DNA damage repair, reduction of apoptosis, and alteration of drug metabolism. Moreover, MDR is usually relative with increased expression of ATP-binding cassette (ABC) transporters that can pump out intracellular substrate agents[2, 3].

Human ABC transporters are a large and widespread family of transmembrane proteins that transport their substrates across cell membrane. The human genome includes 48 ABC genes, that can be subdivided into 7 subfamilies (from ABCA to ABCG) according to their sequence similarities[4, 5]. The significant contribution of thethree major ABC transporters, including ABCB1 (P-glycoprotein/P-gp/MDR1), ABCG2 (Breast Cancer Resistance Protein/BCRP/MXR), and ABCC1 (Multidrug Resistance Protein 1/MRP1) to MDR in cancer chemotherapy is well documented in vitro studies. These ABC transporters are capable to bind and hydrolyze ATP to provide energy for pumping out intracellular chemotherapeutic agents, leading to the reduced efficiency of therapeutic treatment[6–8]

ABCB1 is the most extensively studied MDR transporter. It is expressed in many normal tissues like liver, kidney, muscle, and brain, and is associated with the excretion of toxic metabolites and tissue defense function[9]. In cancer, ABCB1 mediates the effectively efflux of its substrate chemotherapeutic agents, including doxorubicin (Dox), vincristine, and paclitaxel, out of the cells. Compared to ABCB1, the other major MDR transporters also share similar substrate specificity[10]. Common ABCG2 substrate anticancer drugs include mitoxantrone (MX), Dox, topotecan, whereas common ABCC1 substrate chemotherapeutic agents are vincristine and Dox[2, 11]. As MDR is usually related to the increased expression of MDR transporters, the combination of chemotherapeutic drugs with agents capable of inhibiting ABC transporters represent a logical and attractive strategy to overcome MDR in cancer chemotherapy.

The *KRAS* gene is an extensively studied oncogene that encodes a small GTPase transducer protein KRAS to regulate intracellular signal transduction, thereby controlling cell growth and differentiation[12]. The KRAS protein belongs to the family of guanosine triphosphate (GTP) binding proteins, which regulates the transfer information by switching between the GTP-bound states and guanosine diphosphate (GDP)-bound states. In the GTP-bound state, KRAS is able to activate its downstream effector proteins like RAF and PI3K kinases to accelerate cell proliferation. Hyperactivation of KRAS signaling, which is usually caused by mutations of the *KRAS* gene, is commonly observed in cancer to drive uncontrolled cell proliferation and metastasis[13, 14].The KRAS G12C mutation is one of the most extensively studied oncogenic driver mutations. G12C is a single point mutation, where glycine is substituted by cysteine at codon 12[15–17]. This substitution favors the activated GTP-bound state of KRAS and constitutive activation of downstream signaling pathway to drive oncogenesis. MRTX849 is an orally administered and mutation-selective covalent inhibitor of KRAS G12C with potent anticancer activity and it blocks KRAS signaling by irreversibly binding to the mutant KRAS G12C and locking it into an inactive GDP-bound state[18, 19]. In clinical studies, MRTX849 has demonstrated excellent anticancer efficacy and safety profile in patients with non-small cell lung cancer and colorectal cancer harboring the KRAS G12C mutation[20–22].

We previously reported the reversal of MDR by several molecular targeted tyrosine kinase inhibitors[23–25]. However, nothing is known about the effect of KRAS inhibitor on MDR. Herein, we aimed at investigating the reversal ability of MRTX849 to ABC transporters-mediated MDR.

## Materials and methods

### Chemicals and reagents

The following drugs/chemicals, 3-(4,5-Dimethylthiazol-yl)-2,5-diphenyltetrazolium bromide (MTT), Dox, paclitaxel, MX, topotecan, cisplatin, Rho 123, verapamil (VRP), fumitremorgin C (FTC), were purchased from Sigma-Aldrich (St. Louis, MO, USA). MRTX849 was obtained from MedChemExpress (New Jersey, NJ, USA). Antibodies against ABCB1, AKT, p-AKT, ERK, and p-ERK, GAPDH were obtained from Santa Cruz Biotechnology Inc. (Paso Robles, CA, USA). DMEM and RPMI 1640 medium were purchased from Gibco BRL (Gaithersburg, MD, USA). SYBR Green qPCR Master Mix was obtained from ExCell Bio (Shanghai, China).

### Cell lines and cell culture conditions

The cell lines used in this study were as follows: the human breast cancer cell line MCF-7 and its ABCB1-overexpressing resistant cell line MCF-7/adr induced by doxorubicin (DOX); the human oral epidermoid carcinoma cell line KB and its ABCB1-overexpressing resistant cell line KBv200 induced by vincristine (VCR);the human colon carcinoma cell line S1 and its ABCG2-overexpressing resistant cell line S1-M1-80 induced by mitoxantrone (MX); human embryonic kidney cell line HEK293 and their stably transfected cell line HEK293/vector (transfection with vector plasmid), HEK293/ABCB1 (transfection with ABCB1 plasmid)and HEK293/ABCG2 (transfection with ABCG2 plasmid) [26, 27].All cell lines were tested negative for mycoplasma contamination. The cell lines were cultured in RPMI-1640 medium (KB and KBv200) or DMEM medium (other cells) with 10% FBS, 1% penicillin and 1% streptomycin. These cells were culture at 37 °C, 5% CO_2_.

### Cell viability and MDR reversal experiments in vitro

The anticancer activity and MDR reversal ability of MRTX849 in vitro were evaluated by MTT assay as previously described[28]. Briefly, about 6000 cells growing in logarithmic phase were seeded into 96-well plates with 180 μL medium per well and incubated overnight. Subsequently, different drugs (20 μL) were added to produce the desired concentration. MRTX849 was tested at a concentration range of 0–16 μM alone for its cytotoxicity. To detect the MDR reversal activity of MRTX849, cells were cultured with MRTX849, Verapamil (VRP, an inhibitor of ABCB1 protein) or Fumitremorgin C (FTC, an inhibitor of ABCG2 protein) for 1 h first, which was followed by the addition of the conventional anticancer drugs at different concentrations for another 68 h. At the end of the drug incubation, adding MTT-medium (5 mg/mL, 20 μL) to each well to incubate for 4 h. Subsequently, discarding all medium and adding 120 μL DMSO to each well. Finally, the absorbance was detected by enzyme reader at 570/630 nm. The IC50 (concentrations inhibiting growth by 50%) was calculated by the Bliss method. The relative fold of drug resistance was calculated by dividing the IC50 value of resistant cells by that of sensitive cells. The MDR reversalactivity of MRTX849 in MDR cells was expressed as the IC50 value of anticancer drugs in the absence of MRTX849 divided by that in the presence of MRTX849.

### MDR xenograft tumor models

To verify the reversal effect of MRTX849 in vivo, two kinds of MDR xenograft tumor models were established in athymic nude female mice (weight: about 15 g; age: 4–6 weeks) (Guangdong Medical Experimental Animal Center) as described previously[29]. Due to the lack of important parameters in the sample size estimation based on efficacy test, we used the law of diminishing returns to choose the sample size estimation for animal study. According to the formula, we calculated that the sample size of each group should be 6. But taking into account losses caused by model failures, animal deaths and so on, the increase was 10–20%[30, 31].For safety and feasibility considerations, the concentrations of MRTX849 used were chosen based on reported animal and clinical studies[20, 32]. The study was conducted according to the guidelines of the Declaration of Helsinki, and approved by the Animal Ethics Committee of Sun Yat-sen University Cancer Center (No. L102042021010A).

KBv200 cells (1 × 10^7^ cells/200 μl/mouse) or MCF-7/adr cells (1.5 × 10^7^ cells/200 μL /mouse) at logarithmic growth phase were inoculated subcutaneously in nude mice to establish the MDR tumor xenografts at different time points. When the average diameter of tumor reached about 0.5 cm, mice were randomly divided into four treatment groups: (1) normal saline control group (p.o., 10 mg/kg, q3d × 4); (2) MRTX849 alone group (30 mg/kg p.o., q3d × 4); (3) paclitaxel alone group (18 mg/kg i.p., q3d × 4); (4) paclitaxel (18 mg/kg i.p., q3d × 4) + MRTX8498 (30 mg/kg p.o., q3d × 4). MRTX849 was given orally 1 h before paclitaxel injection. Upon the commencement of drug treatment, the body weight of the experimental mice and the long and short diameter (A and B) of each tumor were recorded every 3 days. The tumor volume (V) was estimated according to the following formula: V = (π/6) [(A + B)/2]^3^. Tumor growth curve was obtained by plotting the tumor volume against number of days after tumor implantation. The experiments were terminated when the average tumor weight reached more than 1 g. At termination, all mice were anesthetized by carbon dioxide and killed, and the tumor xenografts was removed and weighed. The tumor growth inhibition rate (IR) was calculated by the formula: tumor inhibition rate (%) = (1—average tumor weight of the experimental group / average tumor weight of the control group) × 100%. The drug toxicity was reflected by the change in mice body weight after drug treatment.

### Dox and Rho 123 accumulation assays

The effect of MRTX849 on cellular accumulation of the fluorescent drug (Dox) or dye (Rho 123) was evaluated by flow cytometry as described previously[33]. Briefly, drug resistant and sensitive cells were seeded in 6-well plates (5 × 10^5^ cells/well) for 24 h. Different concentrations of MRTX849 (0 μM, 0.25 μM, 0.5 μM, 1 μM) or 10 μM VRP (specific ABCB1 inhibitor as positive control) were added to incubate for 3 h. Afterwards, the fluorescent drug (Dox at final concentration of 10 μM) or dye (Rho 123 at final concentration of 5 ug/mL) was added respectively and incubated for an additional 3 h or 30 min, followed by washing with PBS. At last, cells were collected in cold PBS (containing 10 μM VRP) and then measured by flow cytometry.

### Rho 123 efflux assay

To assess the effect of MRTX849 on ABCB1 efflux activity, Rho 123 efflux assay was performed as described previously [34]. Briefly, cancer cells were seeded into 6-well plates (5 × 10^5^ cells/well) for 24 h at 37 °C. After pre-treatment with Rho123 at a final concentration of 5 ug / ml for 30 min, excess Rho 123 was washed out with PBS. Subsequently, cells were cultured with or without MRTX849 at a final concentration of 1 μM. Finally, collecting cells at different time points for flow cytometry analysis.

### ABCB1 ATPase activity assay

The vanadate-sensitive ATPase activity of ABCB1 was evaluated by Corning Gentest ATPase assay (BD Biosciences, San Jose, CA, USA) in cell membrane preparation harvested from ABCB1-overexpressing High-Five insect cells (cat.no.453270) based on manufacturer’s instructions [35]. MRTX849 (0.01 – 5 µM) was incubated with ABCB1-overexpressing cell membrane (100 µg/mL protein) with or without sodium orthovanadate (1.2 mM) in an ATPase assay buffer at 37 °C for 5 min. Next, ATPase hydrolysis was started by adding 12 mM Mg-ATP (total reaction volume was 60 µL). After a further 10-min incubation, the reaction was ended up after adding 10% sodium dodecyl sulfate solution (30 µL). Detecting the release of inorganic phosphate by calculating the absorbance at 800 nm, and drawing the phosphate standard curve for quantification. The ABCB1 ATPase activity stimulated by MRTX849 was defined as the difference in inorganic phosphate released from ATP in the absence and presence of sodium orthovanadate.

### Photo-affinity labeling of ABCB1 with [.^125^I]-iodoarylazidoprazosin (IAAP)

Crude membrane from High Five insect cells overexpressing ABCB1 (50 ng) was incubated with different concentrations of MRTX849 (0–5 nM) in 50 mM Tris–HCl (pH7.5) for 5 min. Afterwards, [^125^I] IAAP was added under subdued light and the reaction mixture was incubated at room temperature for another 5 min and then irradiated by UV light at 365 nm on ice. The labeled ABCB1 was immunoprecipitated using the ABCB1-specific antibody C219 (Enzo Life Sciences, Farmingdale, NY, USA). The samples were then subjected to SDS-PAGE using a 7% Tris–acetate NPAGE gel, dried on a glue dryer and exposed to Bio-Max MR film (Eastman Kodak Co., Rochester, NY) overnight at -80℃.Radioactivity incorporated into the ABCB1 protein bands was quantitatively analyzed by the STORM860 Phosphorimager system.

### Western blot assay

The protein expression level was measured by Western blotting as described previously [36]. Briefly, cells were treated with MRTX849 at different concentrations, and then collected and lysed at different time points. The protein samples were quantified using the BCA protein detection kit (Pierce, Rockford, IL). Equal amounts (20 µg) of protein samples were added to the SDS-PAGE gel and transferred to the PVDF membranes. After sealing in 5% skimmed milk for 1 h, the membranes were incubated with the primary antibody and the secondary antibody, and finally visualized with developer, fixer and X-ray film. GAPDH was used as a reference.

### RNA extraction and qPCR

ABCB1 mRNA expression level was measured by qPCR assay as described previously [37]. Briefly, cells were incubated in 6-well plates overnight and then exposed to MRTX849 at a range of different concentrations (0 μM, 0.25 μM, 0.5 μM, 1 μM, and 10 μM) or time points (0 h, 24 h, 48 h, and 72 h). After drug incubation, cells were collected and total RNA was extracted by the RNeasy Mini Kit (QIAGEN) using standard RNA extraction method. cDNA was synthesized from the total RNA using the Reverse Transcription Ace qPCR RT kit (QIAGEN). qPCR was performed with the SYBR Green qPCR Master Mix. Specific primers for ABCB1 and GAPDH are listed below: 5′-CCCATCATTGCAATAGCAGG-3′(forward) and 5′-GTTCAAACTTCTGCTCCTGA-3′(reverse) for ABCB1, 5′-CTTTGGTATCGTGGAAGGA-3′ (forward) and 5′-CACCCTGTTGCTGTAGCC-3′ (reverse) for GAPDH respectively. The value of GAPDH expression level was used as a control to evaluate that of ABCB1. Relative quantification of the samples was calculated by the 2^–ΔΔCt^ method.

### ABCB1 expression on cell surface by flow cytometry

Flow cytometry was performed to measure the expression of ABCB1 protein on cell surface as early described[27]. After incubating with or without MRTX849 for 48 h, MDR cells were collected and made into single-cell suspension (5 × 10^5^ cells/100 ul) followed by incubating with flow cytometry antibodies against human P-gp or mouse IgG2b/κ (negative control) for 45 min at 4 °C with no light. Finally, ABCB1 expression on cell surface was detected by flow cytometry.

### Immunofluorescence

The cells were cultured in a glass substrate confocal culture dish, treated with 1 μM MRTX849 for 48 h, washed with PBS 3 times, and fixed with paraformaldehyde for 15 min. The membrane was permeated with 0.1% Triton X-100 and then sealed with 1% BSA. The primary antibody was incubated overnight and the secondary antibody was incubated for 1 h. Finally, DAPI staining was performed and images were obtained using Zeiss LSM 880 confocal microscope[38].

### Molecular docking

Molecular docking is an effective method widely used in drug discovery that can be used to explore the interaction mode of small molecules in the binding site of a target protein. In this study, molecular docking analysis was used to further reveal the binding mode of MRTX849 to the nucleotide-binding domain (NBD) of ABCB1. The crystal structure of ABCB1 was obtained from the Protein Data Bank (PDB, https://www.rcsb.org/), and the 3D molecular structure of MRTX849 was obtained from PubChem (https://pubchem.ncbi.nlm.nih.gov/). AutoDock Vina was used to perform docking studies, and PyMOL 1.8 was used to visualize and display the results (molecule: MRTX849; ABCB1 PDB: 6C0V).

### Statistical analysis

All experiments were repeated at least three times and the differences were analyzed by Student's *t*-test and statistical significance was defined as p < 0.05.

## Results

### MRTX849 re-sensitized MDR cells overexpressing ABCB1 to anticancer drugs in vitro

**Fig. 1:**
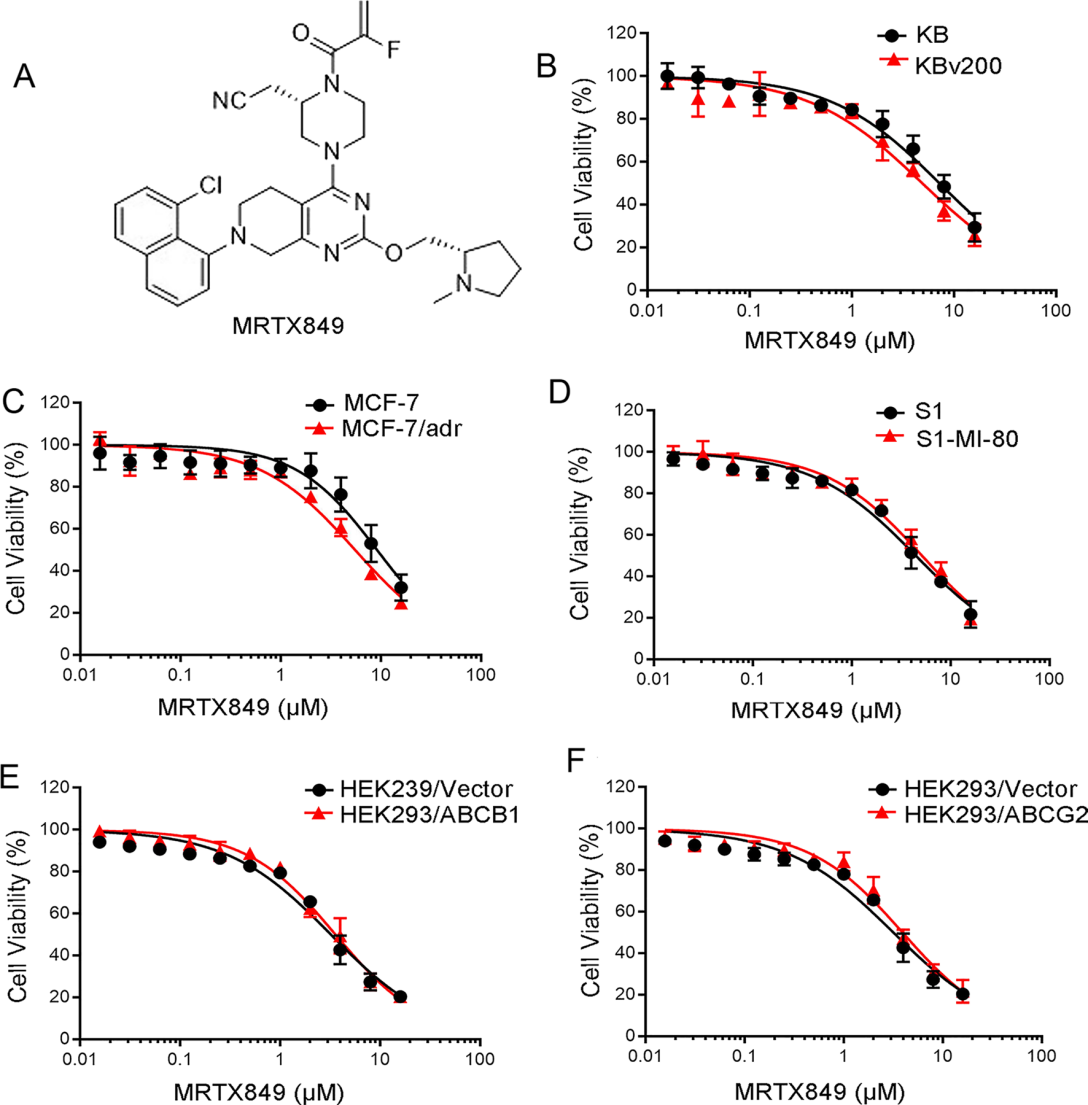
Chemical structure of MRTX849 and cytotoxicity of MRTX849 in cancer cells. **A** Structure of MRTX849. **B-F** Cell viability curves for KB and KBv200 cells, MCF-7 and MCF-7/adr cells, S1 and S1-M1-80 cells, HEK293/Vector and HEK293/ABCB1 cells, and HEK293/Vector and HEK293/ABCG2 cells. All cell lines were incubated with MRTX849 for 72 h. All data are presented as means ± SEM

Before investigating the chemo-sensitization effect of MRTX849, MTT assay was conducted to select drug concentrations without significant cytotoxic effects (Fig. 1A). Our results showed that more than 80% of drug-resistant and sensitive cells survived after treatment with MRTX849 at the concentration under 1 μM (Fig. 1B–F). Therefore, the concentrations of MRTX849 at 0.25 μM, 0.5 μM and 1 μM were chosen for all subsequent experiments.

We next evaluated MDR reversal effect of MRTX849 by calculating the IC50 values of cancer cells treated with different concentrations of MRTX849. In the absence of MRTX849, the IC50 values of drug resistant cells overexpressing ABCB1 (KBv200 and MCF-7/adr cells) to ABCB1 substrate anticancer drugs (paclitaxel and Dox) or drug resistant cells overexpressing ABCG2 (SI-M1-80 cells) to ABCB1 substrate anticancer drugs (MX and topotecan) were much higher than that of the corresponding sensitive cells KB, MCF-7, and S1 (Table 1). Importantly, treatment with MRTX849 remarkably lowered the IC50 values of ABCB1 substrate chemotherapeutic agents in the ABCB1 overexpressing MDR cells, but not in the corresponding sensitive cells. Furthermore, ABCB1-transfected HEK293/ABCB1 cells were much more sensitive to substrate anticancer drugs after treatment with MRTX849. However, MRTX849 did not alter the cytotoxic effect of cisplatin, a non-ABCB1 substrate agent. Moreover, MRTX849 did not exhibit significant MDR reversal effect on ABCG2-mediated MDR cells (Table 1). The above findings illustrated that MRTX849 could selectively improve the response of drug-resistant cells mediated by ABCB1 to substrate anticancer drugs in vitro.

**Table 1:** Effect of MRTX849 on the reversal of MDR mediated by ABCB**1**

Compounds	IC50 ± SD (μM) (fold-reversal)			
	KB		KBv200 (ABCB1)	
Doxorubicin	0.029 ± 0.063	(1.00)	2.091 ± 0.046	(1.00)
+ MRTX849 (0.25 μM)	0.028 ± 0.091	(1.06)	1.503 ± 0.079*	(1.39)
+ MRTX849 (0.5 μM)	0.026 ± 0.076	(1.13)	0.929 ± 0.047**	(2.25)
+ MRTX849 (1 μM)	0.026 ± 0.020	(1.14)	0.331 ± 0.092**	(6.32)
+ Verapamil (10 μM)	0.027 ± 0.068	(1.11)	0.107 ± 0.089**	(19.46)
Paclitaxel	0.002 ± 0.036	(1.00)	0.580 ± 0.056	(1.00)
+ MRTX849 (0.25 μM)	0.002 ± 0.075	(1.00)	0.241 ± 0.080**	(2.41)
+ MRTX849 (0.5 μM)	0.002 ± 0.073	(1.06)	0.165 ± 0.064**	(3.53)
+ MRTX849 (1 μM)	0.002 ± 0.057	(0.96)	0.033 ± 0.038**	(17.81)
+ Verapamil (10 μM)	0.003 ± 0.080	(0.93)	0.027 ± 0.069**	(21.51)
Cisplatin	0.611 ± 0.024	(1.00)	2.579 ± 0.053	(1.00)
+ MRTX849 (1 μM)	0.608 ± 0.035	(1.01)	2.575 ± 0.056	(1.00)

	MCF-7		MCF-7/adr (ABCB1)	
Doxorubicin	0.380 ± 0.020	(1.00)	11.667 ± 0.405	(1.00)
+ MRTX849 (0.25 μM)	0.387 ± 0.062	(0.98)	5.812 ± 0.023**	(2.01)
+ MRTX849 (0.5 μM)	0.401 ± 0.048	(0.95)	2.386 ± 0.042**	(4.90)
+ MRTX849 (1 μM)	0.391 ± 0.093	(0.97)	1.596 ± 0.081**	(7.31)
+ Verapamil (10 μM)	0.376 ± 0.109	(1.01)	0.798 ± 0.012**	(14.61)
Paclitaxel	0.036 ± 0.010	(1.00)	1.070 ± 0.063	(1.00)
+ MRTX849 (0.25 μM)	0.035 ± 0.032	(0.97)	0.629 ± 0.060*	(1.70)
+ MRTX849 (0.5 μM)	0.033 ± 0.038	(0.96)	0.222 ± 0.059**	(4.83)
+ MRTX849 (1 μM)	0.028 ± 0.083	(1.58)	0.068 ± 0.191**	(15.85)
+ Verapamil (10 μM)	0.032 ± 0.072	(1.26)	0.052 ± 0.070**	(20.69)
Cisplatin	5.242 ± 0.075	(1.00)	6.630 ± 0.046	(1.00)
+ MRTX849 (1 μM)	5.172 ± 0.120	(1.01)	6.455 ± 0.154	(1.03)

	S1		S1-MI-80 (ABCG2)	
Mitoxantrone	0.173 ± 0.038	(1.00)	6.497 ± 0.107	(1.00)
+ MRTX849 (0. 25 μM)	0.178 ± 0.065	(0.98)	6.527 ± 0.029	(0.99)
+ MRTX849 (0. 5 μM)	0.161 ± 0.096	(1.08)	6.558 ± 0.030	(0.99)
+ MRTX849 (1 μM)	0.191 ± 0.133	(0.92)	6.578 ± 0.061	(0.99)
+ FTC (2.5 μM)	0.142 ± 0.053	(1.21)	0.533 ± 0.040**	(12.19)
Topotecan	0.250 ± 1.082	(1.00)	8.950 ± 0.288	(1.00)
+ MRTX849 (0.25 μM)	0.274 ± 0.085	(0.92)	9.250 ± 0.097	(0.98)
+ MRTX849 (0.5 μM)	0.241 ± 0.073	(1.02)	9.206 ± 0.098	(0.98)
+ MRTX849 (1 μM)	0.281 ± 0.043	(0.89)	9.104 ± 0.079	(0.99)
+ FTC (2.5 μM)	0.251 ± 0.079	(0.98)	0.753 ± 0.074**	(11.93)
Cisplatin	10.152 ± 0.261	(1.00)	8.864 ± 0.116	(1.00)
+ MRTX849 (1 μM)	9.931 ± 0.221	(1.02)	8.501 ± 0.170	(1.02)

	HEK293/Vector		HEK293/ABCB1	
Doxorubicin	0.061 ± 0.004	(1.00)	1.218 ± 0.081	(1.00)
+ MRTX849 (0.25 μM)	0.059 ± 0.067	(1.03)	0.474 ± 0.074**	(2.57)
+ MRTX849 (0.5 μM)	0.059 ± 0.060	(1.02)	0.315 ± 0.061**	(3.87)
+ MRTX849 (1 μM)	0.061 ± 0.082	(1.00)	0.116 ± 0.067**	(10.47)
+ Verapamil (10 μM)	0.063 ± 0.055	(0.96)	0.075 ± 0.065**	(16.18)
Paclitaxel	0.029 ± 0.001	(1.00)	2.543 ± 0.086	(1.00)
+ MRTX849 (0.25 μM)	0.028 ± 0.071	(1.05)	1.643 ± 0.030*	(1.70)
+ MRTX849 (0.5 μM)	0.028 ± 0.099	(1.02)	0.670 ± 0.052**	(3.79)
+ MRTX849 (1 μM)	0.025 ± 0.049	(1.17)	0.030 ± 0.096**	(8.36)
+ Verapamil (10 μM)	0.026 ± 0.034	(1.09)	0.023 ± 0.182**	(11.19)
Cisplatin	1.840 ± 0.062	(1.00)	2.247 ± 0.109	(1.00)
+ MRTX849 (1 μM)	1.795 ± 0.010	(1.03)	2.148 ± 0.075	(1.05)

The cell viability was measured by the MTT assay. Data are shown as the mean ± SD of at least three independent experiments. The fold-reversal of MDR was calculated by dividing the IC50 value for cells treated with the anticancer agents without MRTX849 by that treated with MRTX849. *P < 0.05, **P < 0.01 vs. control group

### MRTX849 potentiated the antitumor efficacy of paclitaxel in ABCB1-overexpressing tumor xenograft in vivo

**Fig. 2:**
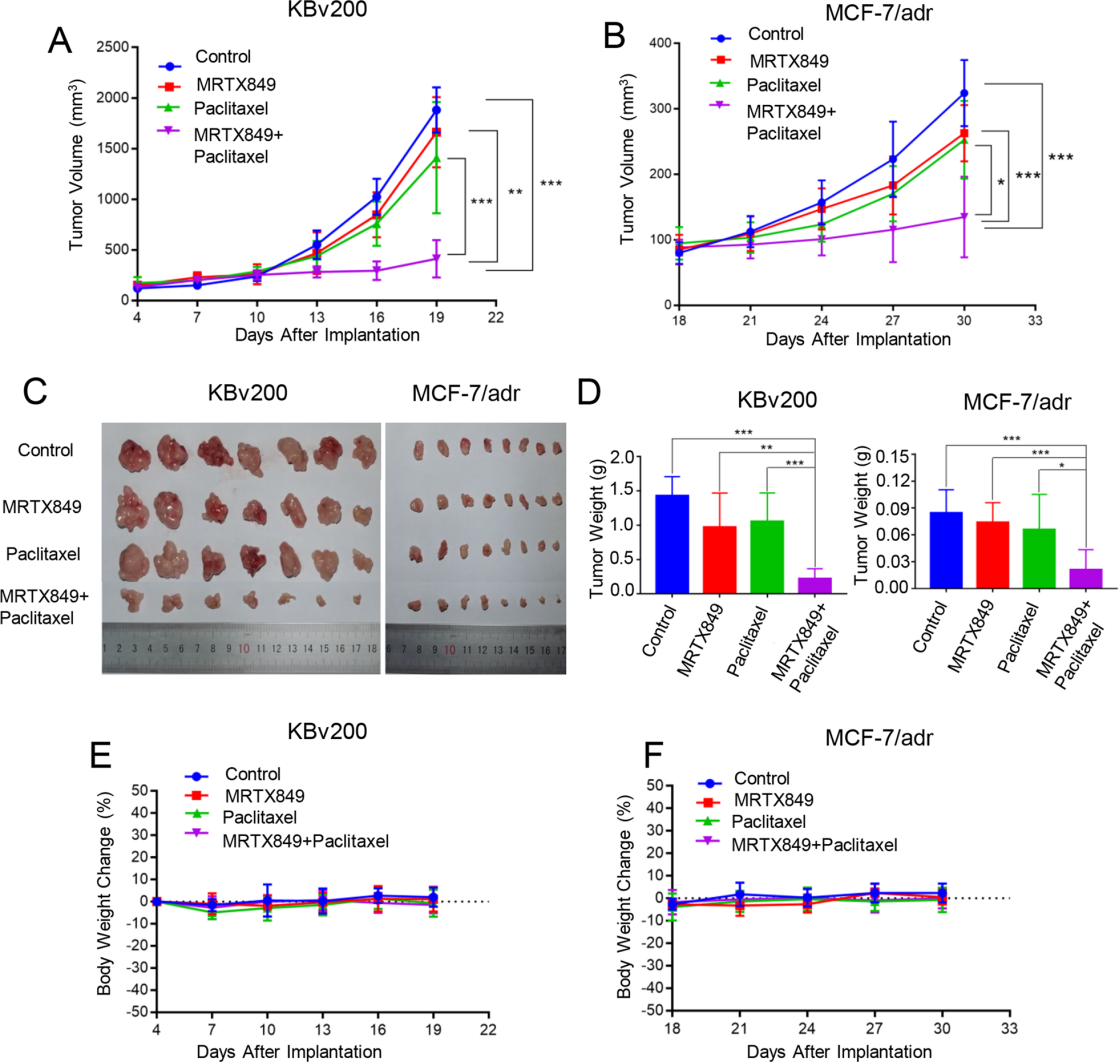
Potentiation of paclitaxel anticancer activity by MRTX849 in ABCB1-overexpressing MDR tumor xenograft models in vivo. **A**, **B** The change in tumor volume in tumor xenograft derived from KBv200 cells or MCF-7/adr cells. **C**, **D** Tumors were stripped and weighed at the end of the experiment. **E**, **F** Body weight of mice were measured every 3 days, and shown as the average percentage changes

To further confirm the MDR reversal ability of MRTX849 in vivo, two ABCB1-overexpressing tumor xenograft models were established by KBv200 or MCF-7/adr cells in female nude mice, respectively. In both ABCB1-mediated MDR tumor xenograft models, no notable difference was observed in tumor growth among nude mice treated with normal saline, MRTX849 or paclitaxel alone. The results illustrated that the tumor xenograft was resistant to paclitaxel and MRTX849 alone and did not exhibit appreciable antitumor effect (Fig. 2A–D). Nevertheless, combination of paclitaxel and MRTX849 resulted in greater suppression of tumor growth than other treatment groups (p < 0.05) (Fig. 2A–D). Moreover, no apparent weight loss or drug-related deaths were observed at the combination group, suggesting that no additional toxicity was caused by the drug combination (Fig. 2E, F).

### MRTX849 augmented the accumulation of Dox and Rho 123 in ABCB1-overexpressing cells

**Fig. 3:**
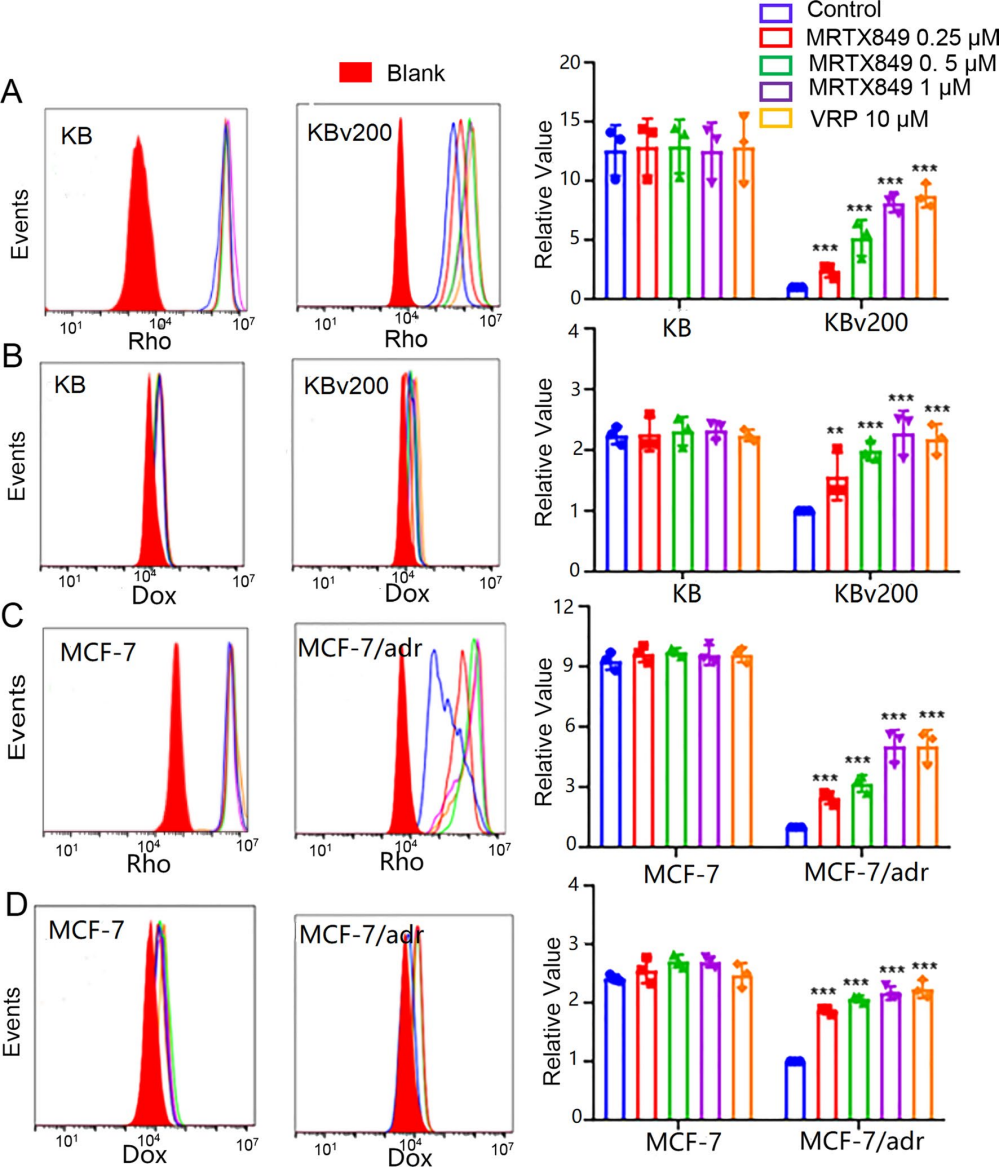
Effect of MRTX849 on intracellular accumulation of substrate agents in ABCB1-overexpressing MDR cells and sensitive cells. **A**-**D** The intracellular accumulation of Rho123 in KB, KBv200 cells or MCF-7, MCF-7/adr cells and that of Dox in KB, KBv200 cells or MCF-7, MCF-7/adr cells. Data are shown as fold-change in fluorescence intensity relative to untreated control MDR cells. *P < 0.05, **P < 0.01, *** P < 0.001, compared with control

The results presented above showed that MRTX849 could reverse resistance to ABCB1 substrate chemotherapeutic drugs in MDR cells. To further investigate the underlying mechanism, the accumulation of Dox and Rho 123 in MDR cells and the corresponding sensitive cells were assessed by flow cytometry. Compared with drug-resistant cells, the intracellular accumulation of the fluorescent intensity of Dox and Rho 123 were remarkably higher in the drug sensitive KB and MCF-7 cells (Fig. 3). When the cells were pre-treated with different concentrations of MRTX849 or VRP, the intracellular accumulation of Dox and Rho 123 in MDR cells overexpressing ABCB1 was increased in a dose-dependent manner. In contrast, similar effect did not occur in sensitive cells.

Collectively, these results demonstrated that MRTX849 could sensitize ABCB1-mediated MDR cells to chemotherapeutic agents though enhancing the intracellular concentration of chemotherapeutic agents.

### MRTX849 inhibited the efflux of Rho 123 by blocking substrate binding to ABCB1 in ABCB1-overexpressing MDR cells

**Fig. 4:**
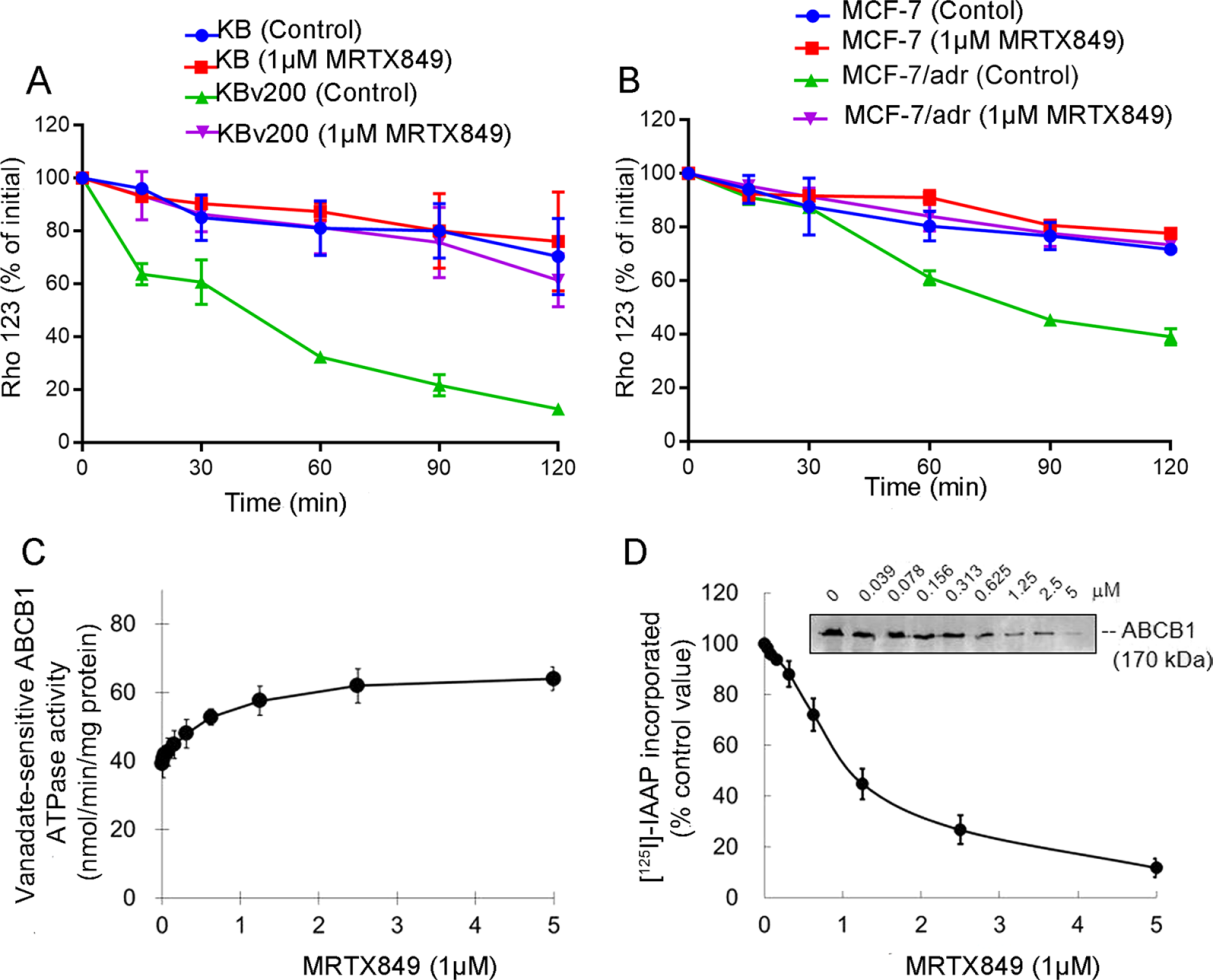
Effect of MRTX849 on the efflux of Rho 123, ABCB1 ATPase activity and [^125^I]-IAAP photoaffinity labeling of ABCB1. **A**, **B** Rho 123 efflux at different time points was monitored in KB, KBv200 cells or MCF-7, MCF-7/adr cells after treatment with or without 1 μM MRTX849. **C** Effect of MRTX849 on ABCB1 ATPase activity. **D** MRTX849 competed for photolabeling of ABCB1 by [^125^I]-IAAP

The above data indicated that MRTX849 was able to elevate the accumulation of fluorescent substrates in ABCB1-mediated MDR cells. We next carried out drug efflux assay to further confirm whether the reversal effect was a result of the repression efflux activity by MRTX849. In the drug efflux assay, the residual amount of intracellular Rho 123 at different time points after an initial drug incubation with or without 1 µM MRTX849 was measured by flow cytometry. Our data showed that in the absence of MRTX849, the efflux of Rho 123 from ABCB1-overexpressing MDR cells was observably more than that from the corresponding sensitive cells (Fig. 4A, B). Importantly, the remaining intracellular Rho 123 level was markedly increased in drug resistant cells after co-incubation with 1 µM MRTX849. In contrast, no apparent alteration of Rho 123 efflux was observed in the sensitive cells with or without MRTX849 (Fig. 4A, B). These results suggested that MRTX849 could inhibit the drug efflux ability in ABCB1-overexpressing MDR cells.

The ABC transporter substrates stimulate the hydrolysis of ATP and the energy released is used for ABC proteins to pump out intracellular chemotherapy agents [39]. To examine whether MRTX849 influenced ABCB1 ATPase activity, we measured the vanadate-sensitive ATPase activity of the transporter exposed to different concentrations of MRTX849. Our results showed that MRTX849- stimulated activity of ABCB1 ATPase was dose-dependent and the stimulated ABCB1 ATPase activity reached a plateau near 62 nmol/min/mg protein at MRTX849 concentrations above 2.5 μM (Fig. 4C).

[^125^I]-IAAP is an ^125^I-labeled arylazide analogue ofprazosin, a ABCB1 substrate, which is often used for identifying the binding sites of the ABC transporters interacting with substrates. Previous reports have demonstrated that substrates or inhibitors of ABCB1 would compete with [^125^I]-IAAP for photolabeling of the transporter. In order to identify the possible interaction of MRTX849 with the substrate-binding regions of ABCB1, crude membrane from ABCB1-overexpressing High Five insect cells was incubated with [^125^I]-IAAP and increasing concentration (0–5 μM) of MRTX849. MRTX849 was found to significantly inhibit the [^125^I]-IAAP photolabeling of ABCB1 in a concentration-dependent manner. The concentration at which 50% inhibition of the [^125^I]-IAAP photoaffinity labeling of ABCB1 by MRTX849 was about 1 μM (Fig. 4D). Therefore, MRTX849 may compete with the transporter substrates to interact with the substrate binding sites of ABCB1, leading to suppress drug efflux and increase intracellular accumulation of chemotherapeutic drugs.

### MRTX849 had no effect on expression and cellular localization of ABCB1 in MDR cells

**Fig. 5:**
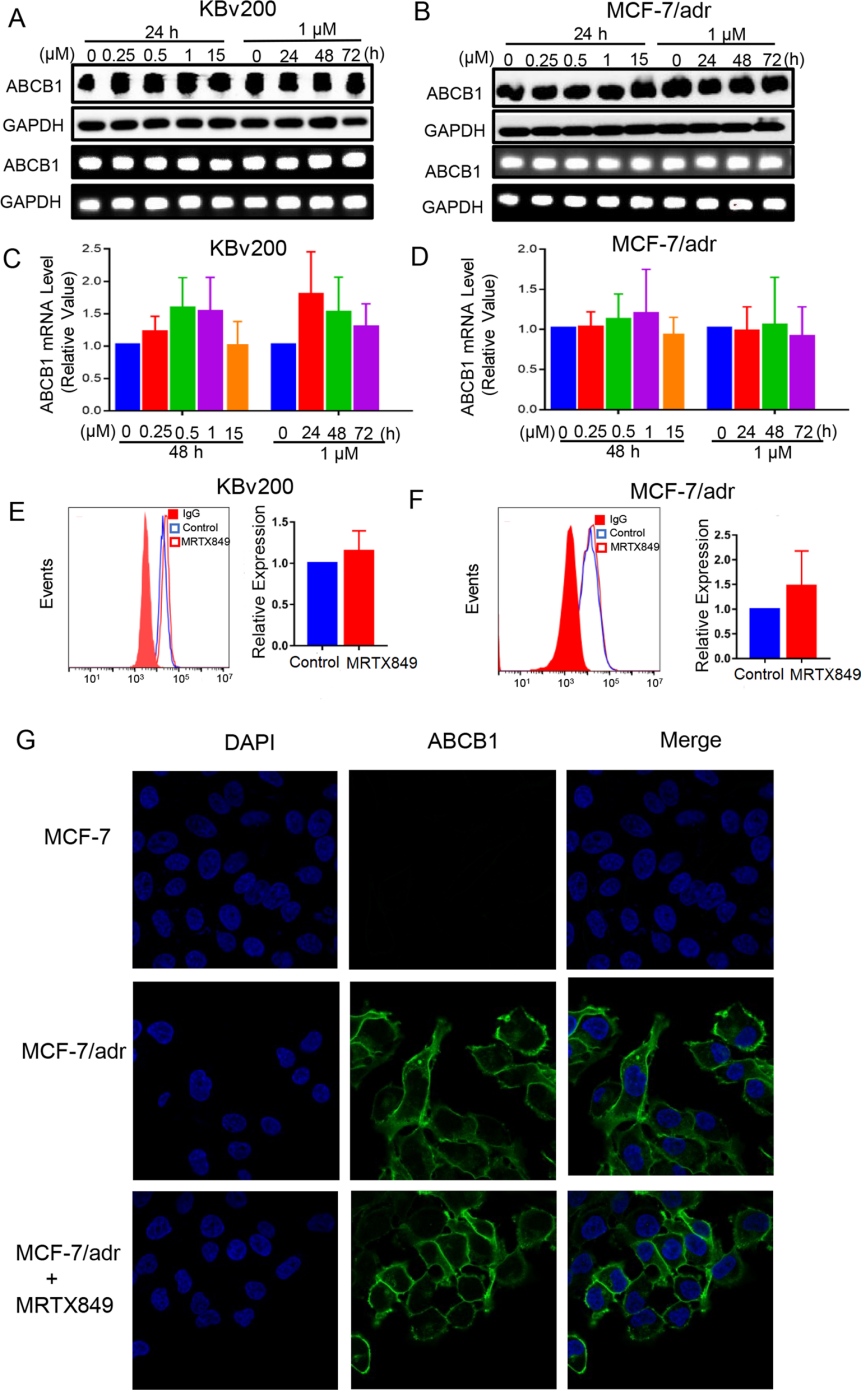
Effect of MRTX849 on ABCB1 expression level in MDR cells. **A**, **B** ABCB1-overexpressing MDR cells were incubated with MRTX849 at various concentrations or time points. MRTX849 had no effect on ABCB1 protein level measured by Western blot assay. **C**, **D** MRTX849 had no effect on ABCB1 mRNA expression measured by qPCR assay. **E**, **F** MRTX849 did not change ABCB1 expression on cell surface measured by flow cytometry. **G** The subcellular localization pattern of ABCB1 was evaluated using confocal laser scanning microscopy. ABCB1 (green) and nuclei (DAPI, blue) were visualized

The reversal of MDR in ABCB1-overexpressing cells may be mediated by downregulation of the ABCB1 transporter protein or inhibition of its drug efflux activity [40]. Western blot and qPCR analyses were conducted to verify whether MRTX849 changed the expression of ABCB1 in MDR cells. The results showed that MRTX849 did not alter the ABCB1 expression at both mRNA and protein level in ABCB1-overexpressing MDR cells (Fig. 5A–D). To investigate if MRTX849 had effect on the intracellular localization of ABCB1, the cell surface expression of ABCB1 in ABCB1-overexpressing MDR cells was evaluated with flow cytometry and immunostaining after treatment with or without 1 μM MTRX849. As expected, ABCB1 was highly expressed on the cell surface of the untreated MDR cells. Upon MRTX849 (1 μM) treatment, the results showed the expression level of ABCB1 at the cell surface did not change appreciably (Fig. 5E, F), and MRTX849 did not alter the plasma membrane localization of ABCB1(Fig. 5G).

### MRTX849 did not affect the phosphorylation of AKT or ERK at MDR reversal concentrations

**Fig. 6:**
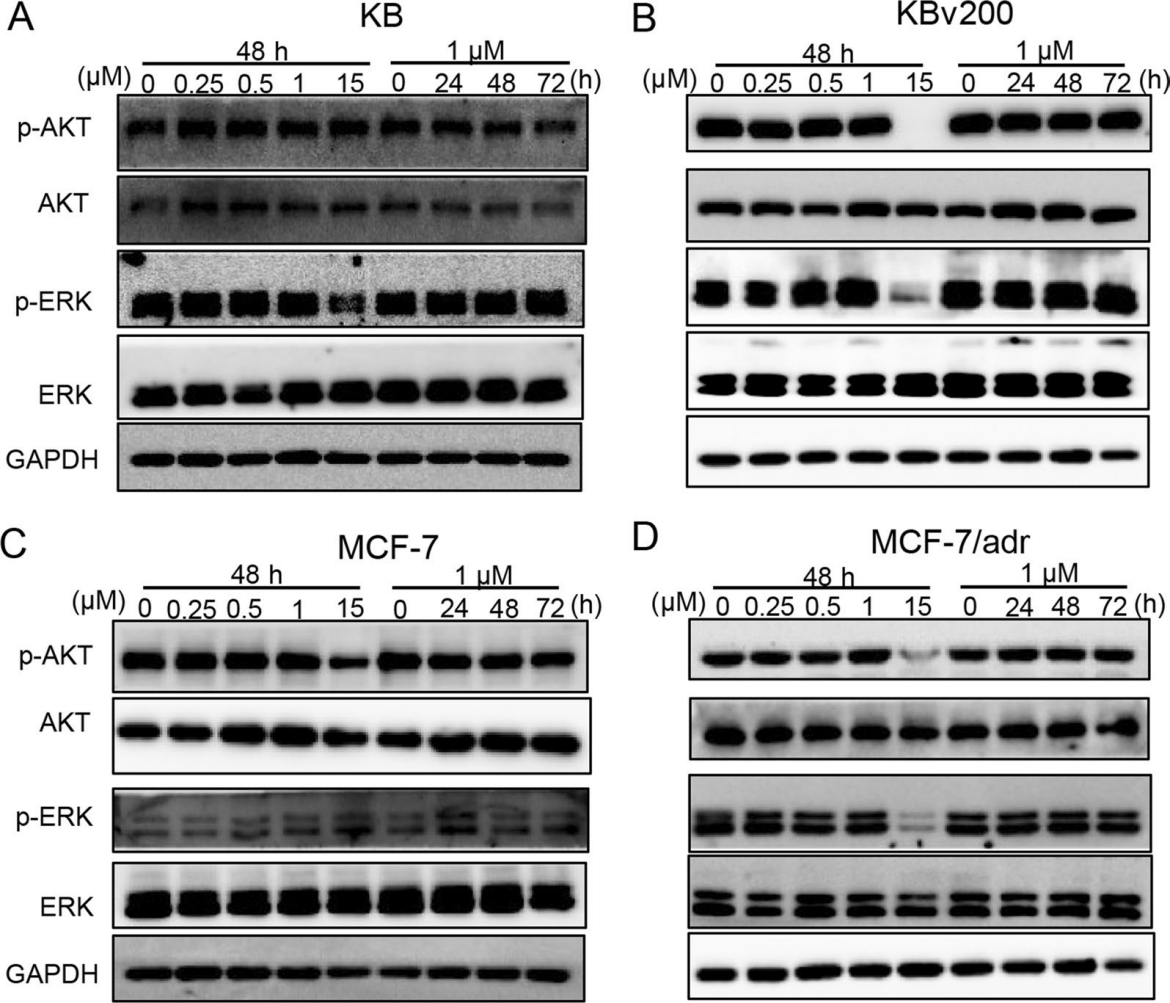
Effect of MRTX849 on the activation of AKT and ERK in cancer cells. **A**-**D** The total and phosphorylation protein level of AKT and ERK were detected by Western blot analysis. KB, KBv200, MCF-7 and MCF-7/adr cells were treated with different concentrations of MRTX849. MRTX849 at 15 μM was used as positive control for the blockade of AKT or ERK phosphorylation. GAPDH was used as the loading control

Previous studies showed that blockade of the AKT and ERK pathways could potentiate anticancer effect of chemotherapy drugs[41, 42]. Therefore, the phosphorylation of AKT and ERK in both drug resistant cells and the corresponding sensitive cells was detected after treatment with MRTX849 at different concentrations. As positive control, MRTX849 at 15 μM was known to inhibit AKT or ERK phosphorylation (Fig. 6). However, when the ABCB1-overexpressing cells were treated with MRTX849 at the effective MDR reversal concentration (1 μM), there was no appreciable change of AKT/ERK phosphorylation (Fig. 6), thus suggesting that reversal of ABCB1-mediated MDR by MRTX849 in drug resistant cells was not relative with AKT or ERK phosphorylation blockages.

### Protein docking analysis

**Fig. 7:**
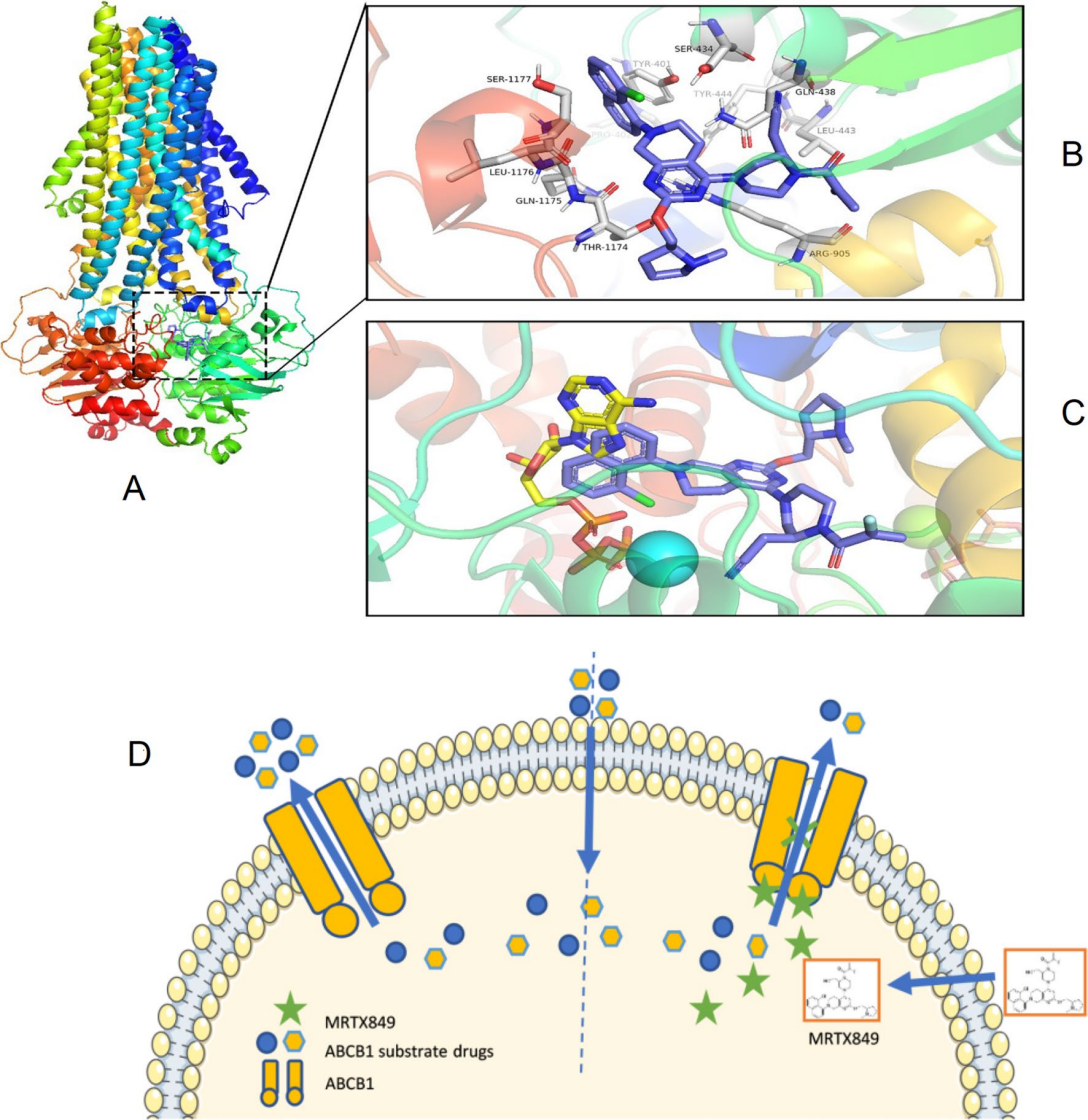
Schematic diagram of the ABCB1 binding with MRTX849 and the reversal of ABCB1-mediated MDR by MRTX849. **A** Overview of the patterns of the MRTX849 binding with ABCB1. **B** MRTX849 was depicted as purple, the white indicates amino acids residues of nucleotide-binding domains (NBD) in ABC transporters. **C** The comparison of MRTX849 and ATP in binding with ABCB1, ATP was displayed as yellow, MRTX849 was depicted as purple. **D** MRTX849 competed with the transporter substrate drugs to interact with the substrate-binding sites, thus increasing cellular accumulation of the drugs and circumvention of MDR

Protein docking analysis showed that MRTX849 can interact well with the nucleotide-binding domain of ABCB1 with a binding energy as low as -10.5 kcal/mol. ABCB1 drives conformational changes to squeeze toxic molecules and drugs out of cells through ATP binding and hydrolysis. The docking results speculate that MRTX849 can effectively compete for ATP to occupy the nucleotide-binding domain of ABCB1 and inhibit its binding to ATP, thereby enhancing the cellular accumulation and anticancer efficacy of the drug (Fig. 7A–C).

## Discussion

MDR remains a determinant factor for failure in cancer chemotherapy. Among the various mechanisms leading to MDR, upregulation of ABC drug efflux transporters is widely observed in drug resistant cells from different cancer types[43, 44]. The overexpression of ABC transporters effectively improves efflux function and reduces intracellular accumulation of chemotherapeutic agents to mediate drug resistance[45].The induction of ABC transporters is recognized as an early event in the development of chemoresistance[46].In order to reverse tumor MDR mediated by ABC transporters, enormous research effort has been made to develop ABC transporter modulators that could suppress the drug efflux activity of MDR cancer cells. In recent years, some tyrosine kinase inhibitors (TKIs) (such as imatinib, dasatinib, gefitinib, erlotinib, lapatinib, sunitinib, sorafenib) have been reported to interact with ABC transporters. Although experimental studies have shown that these reversal agents can reverse tumor MDR, most previously studied inhibitors of ABC transporters are undergoing preclinical or clinical trials.[38, 47–49].As overexpression of ABC transporters in MDR cancers is strongly related to poor prognosis and dismal survival in cancer patients, suppressing ABC transporter function remains a viable strategy to potentiate anticancer efficacy of chemotherapeutic drugs and to reverse MDR.

KRAS is a kind of GTP-binding signaling proteins that takes a critical part in regulating cancer cell proliferation, differentiation, survival and migration [50]. *KRAS* gene can be in a normal state without mutation (known as the wild type) or the abnormal state of mutation (known as mutant type), and the mutated *KRAS* gene would stimulate and promote the proliferation and metastasis of malignant cancer cells [51, 52]. KRAS G12C is the most frequent mutation in pancreatic and colorectal cancer. MRTX849 is a potent, highly selective, and covalent KRAS G12C inhibitor, currently under active clinical investigation[53, 54]. Apart from the investigation of MRTX849 in KRAS-dependent tumors there has been no report about the role of MRTX849 in MDR tumors. Here, the reversal of ABCB1-mediated MDR by MRTX849 was investigated both in vitro and in vivo.

MTT assay was carried out to measure cytotoxic effect of MRTX849 and its circumvention of MDR in ABCB1-overexpressing cancer cell lines. As shown in Fig. 1, there is no significant cytotoxicity of MRTX849 for cells with non-KRAS mutations or even non-KRAS(G12C) mutations, such as KB, KBv200, MCF-7, MCF-7/adr cells used in this experiment. One study evaluated the growth-inhibitory activities of MRTX849C, in seven NSCLC cell lines carrying KRAS G12 mutations (three G12C and four non-G12C), and all non-G12C cell lines were resistant as expected, largely consistent with our results[55]. Since MRTX849 did not appreciably affect cell viability at concentration < 1 μM, it was tested at 0.25 μM, 0.5 μM and 1 μM for subsequent MDR reversal experiment. Our data indicated that MRTX849 specifically re-sensitized ABCB1-mediated MDR cells to transporter substrate agents including Dox and paclitaxel, but not in ABCG2-overexpressing cells or the corresponding sensitive cells. More importantly, MRTX849 was also found to reverse the drug resistance to paclitaxel in two ABCB1-overexpressing MDR xenografts in vivo.

The underlying mechanism for the MDR reversal by MRTX849 was also evaluated systematically. Our data indicated that MRTX849 improved intracellular ABCB1 substrates accumulation in MDR cells as a result of restraining efflux function of ABCB1 transporters, but not in drug-sensitive cells. Since the energy for drug efflux was attained from ATP hydrolysis by ATPase, we next tested if MRTX849 had effect on the ATPase activity. At concentration below 1 mM that is sufficient to overcome drug resistance, MRTX849 stimulated the ATPase activity of ABCB1 and it also competed with [^125^I]-IAAP for the photo-affinity labeling of ABCB1. Therefore, MRTX849 may be a competitive inhibitor of ABCB1. We next measured the change of ABCB1 expression after different treatments with MRTX849 by using WB and qPCR assays. However, no significant difference of ABCB1 expression was found in groups treated with or without MRTX849. MRTX849 also had no change on predominantly cell membrane localization of ABCB1 in the MDR cancer cell lines. Moreover, when tested at the effective MDR reversal concentrations, MRTX849 did not appreciably affect the activation of AKT and ERK signaling pathways.

Chemotherapy is also the main treatment for lung cancer in advanced stage. The substrate drugs used in this study mainly included doxorubicin, paclitaxel, and cisplatin. In the chemotherapy regimen of non-small cell lung cancer, platinum drugs can be combined with vinorelbine, paclitaxel, and gemcitabine as the first-line treatment regimen[56] Our experimental results showed that MRTX849 was the substrate of ABCB1, and could competitively bind to the substrate binding site of ABCB1, thereby inhibiting the binding of other chemotherapeutic drugs with ABCB1, reducing drug efflux, promoting intracellular drug concentration and enhancing chemotherapy effect. Compared to tons of EGFR inhibitors studied as ABCB1 reversal agents, MRTX849 showed good anticancer efficacy and safety in patients with non-small cell lung cancer and colorectal cancer with KRAS G12C mutation in clinical studies[49, 57]. This study suggested that the combined application of MRTX849 and conventional chemotherapy drugs has clinical guiding significance for enhancing the killing effect of anticancer drugs on drug-resistant cells, which is worthy of further research.

## Conclusion

The present study was the first to demonstrate that the specific KRAS G12C inhibitor MRTX849 inhibits the drug efflux function of ABCB1 protein by interacting with the nucleotide binding domain of ABCB1, thereby increasing intracellular anticancer drug concentrations and overcoming MDR (Fig. 7). While MRTX849 has attracted a lot of attention as a specific KRAS G12C inhibitor for the treatment of KRAS-dependent tumors, this study revealed that MRTX849 may also be applied in combination with conventional chemotherapeutic agents for overcoming MDR. Further studies are advocated to establish the usefulness of MRTX849 for clinical circumvention of MDR in refractory cancer patients.

## Data Availability

Data used in current studies are available from corresponding authors upon reasonable request. The data are not publicly available due to patient medical information.

## Abbreviations

MDR: Multidrug resistance
ABC: ATP-binding cassette
Dox: Doxorubicin
MX: Mitoxantrone
VRP: Verapamil
FTC: Fumitremorgin C
IAAP: Iodoarylazidoprazosin
Rho 123: Rhodamine 123
